# Incidental and Intentional Learning of Verbal Episodic Material Differentially Modifies Functional Brain Networks

**DOI:** 10.1371/journal.pone.0080273

**Published:** 2013-11-18

**Authors:** Marie-Therese Kuhnert, Stephan Bialonski, Nina Noennig, Heinke Mai, Hermann Hinrichs, Christoph Helmstaedter, Klaus Lehnertz

**Affiliations:** 1 Department of Epileptology, University of Bonn, Bonn, Germany; 2 Helmholtz Institute for Radiation and Nuclear Physics, University of Bonn, Bonn, Germany; 3 Interdisciplinary Center for Complex Systems, University of Bonn, Bonn, Germany; 4 Max Planck Institute for the Physics of Complex Systems, Dresden, Germany; 5 Department of Neurology, University of Magdeburg, Magdeburg, Germany; University of Michigan, United States of America

## Abstract

Learning- and memory-related processes are thought to result from dynamic interactions in large-scale brain networks that include lateral and mesial structures of the temporal lobes. We investigate the impact of incidental and intentional learning of verbal episodic material on functional brain networks that we derive from scalp-EEG recorded continuously from 33 subjects during a neuropsychological test schedule. Analyzing the networks' global statistical properties we observe that intentional but not incidental learning leads to a significantly increased clustering coefficient, and the average shortest path length remains unaffected. Moreover, network modifications correlate with subsequent recall performance: the more pronounced the modifications of the clustering coefficient, the higher the recall performance. Our findings provide novel insights into the relationship between topological aspects of functional brain networks and higher cognitive functions.

## Introduction

During the last years evidence has accumulated suggesting that an improved characterization of time-variant interactions between different regions within the complex network brain can be achieved with graph-theoretical approaches (see [1]–[6] for an overview). Within this framework a network (or graph) is considered as a set of nodes (or vertices) and a set of links (or edges) connecting the nodes. Functional brain networks can be derived from direct or indirect measurements of neural activity (e.g., electroencephalogram (EEG), magnetoencephalogram (MEG), or functional magnetic resonance imaging (fMRI) data). The sampled brain regions are usually considered as network nodes, and network links represent interactions between pairs of brain regions that can be assessed by evaluating interdependencies (see [7]–[11] for an overview) between their neural activities. The resulting connection schemes can then be characterized by network metrics [12] such as the average shortest path length or the clustering coefficient. The average shortest path length 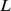 is the mean number of steps along the shortest paths for all possible pairs of nodes. The clustering coefficient 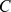 is the mean of the local clustering coefficients of all nodes and quantifies the tendency of nodes to form local clusters. The local clustering coefficient of a node is the fraction of triangles among all connected triples with the node as their center. Large values of both 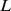 and 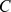 are characteristic for an ordered, lattice-like structure; low values of 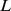 and 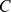 are typically observed for random networks. When analyzing functional brain networks, the clustering coefficient is often interpreted as a measure of the local efficiency of information transfer, and/or of the robustness of the network to deletion of individual nodes. Similarly, the average shortest path length is often interpreted as a measure of the global efficiency of a network to transfer information between nodes [2], [5].

Higher cognitive functions are thought to result from dynamic interactions of distributed brain areas operating in large-scale networks [13]–[18]. Using graph-theoretical approaches, previous studies provided evidence that cognitive tasks–such as motor learning or (n-back) working memory–specifically modify functional brain networks derived from recordings of neural activities (EEG, MEG, fMRI) acquired during the tasks [19]–[35] and that these modifications appear to be associated with task performance [19], [24], [29], [35]. Other studies reported on associations between properties of functional brain networks recorded during a resting state condition and cognitive performance metrics assessed independently during neuropsychological evaluation [36]–[39].

Most of the aforementioned studies investigated cognitive functions that mainly involve prefrontal areas [40] and primary association cortices but only rarely temporolateral neocortex as well as important structures of the mediotemporal lobe, namely hippocampus and surrounding perirhinal cortex and parahippocampal gyri. The lateral and mesial temporal lobe structures are known to be involved in learning- and memory-related processes [41]–[49], and their functionality has frequently been investigated with fMRI [50]–[53], intracranial electrophysiological techniques [54]–[59], or via non-invasively recorded event-related electric potentials or magnetic fields [60].

Here we investigate whether incidental and intentional learning of verbal episodic material differentially modifies functional brain networks and whether modifications are related to subsequent recall performance. To this end, we assess–in a time-resolved manner–global statistical characteristics (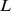 and 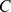) of functional brain networks that we derive from ongoing multichannel EEG data recorded non-invasively from healthy subjects and from epilepsy patients during a neuropsychological test schedule. Chronic epilepsy and its treatment is known to impair cognitive processes and to induce functional reorganization and behavioral compensation [61]–[63]. Investigating both healthy subjects and epilepsy patients thus provides a spectrum of recall performances that is required to study possible relationships between learning-induced network modifications and memory.

## Materials and Methods

### Ethics statement

The study was approved by the local Ethical Committees, and all subjects gave written informed consent.

### Subjects

Thirteen patients (age mean of 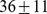 years; 8 females; mean duration of epilepsy 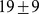 years) with pharmacoresistant epilepsies of suspected temporal or extratemporal neocortical origin as well as twenty healthy controls (age mean of 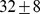 years; 10 females) were included in the study. All patients had been submitted for pre-surgical evaluation at the University of Bonn Epilepsy Program [64]. Seven patients were diagnosed as having a unilateral temporal lobe epilepsy (mesial seizure-onset zone in four patients and lateral seizure-onset zone in three patients). In five patients the seizure-onset zone was located in neocortical structures (central, parietal, parieto-central, parieto-occipital), and one patient was diagnosed as having multiple seizure-onset zones. Surgery was performed in eleven patients (selective amygdalohippocampectomy; extended lesionectomy) and led to complete seizure control in six patients.

### Neuropsychological test schedule and EEG recordings

**Figure 1:**
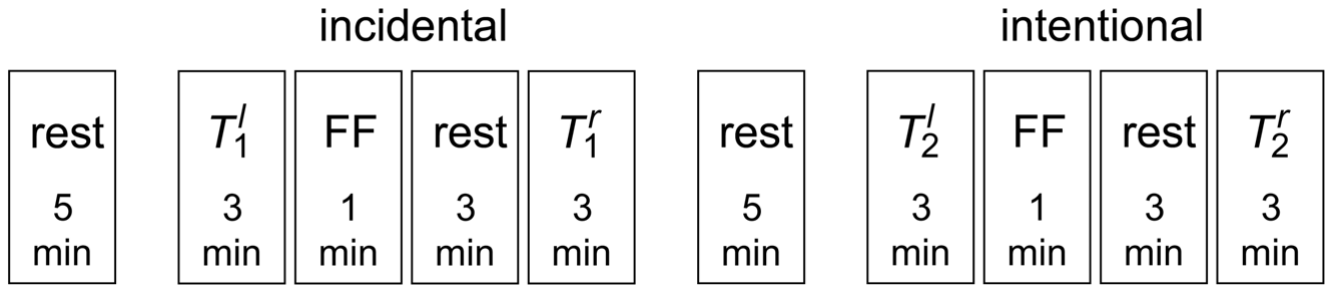
Neuropsychological test schedule. The schedule consists of two blocks of tasks which involve incidental (block 1) or intentional (block 2) learning and retrieval of verbal material. Each block is preceded by a rest phase of 5 minutes duration. Learning tasks (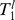 and 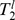, 3 minutes duration) are followed by a figural fluency task (FF, 1 minutes duration) and a rest phase of 3 minutes duration. Retrieval tasks (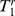 and 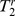, 3 minutes duration) consist of a free recall of words listened to/memorized during the respective learning task.

Before participating in the neuropsychological test schedule, subjects were informed about its general outline. They will be asked to learn a set of words (learning task) and later on, to remember the learned words (retrieval task). They will also be asked to perform an extra *control* task during which they just have to listen to words, however they were not informed about a subsequent retrieval task. The test schedule thus consisted of two blocks of tasks which involved incidental (block 1) or intentional (block 2) learning and retrieval of verbal material (cf. Figure 1):

- Block 1: during a learning task (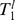) subjects were orally presented 15 highly frequent German nouns (cf. [65]) five times in randomized order. During the retrieval task (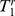) subjects were asked to freely recall words they listened to during 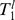.
- Block 2: during a learning task (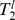) subjects were required to learn 15 highly frequent German nouns (different from those presented during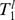) that were orally presented five times in randomized order. During the retrieval task (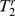) subjects were asked to freely recall words memorized during 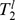.

Learning and retrieval tasks lasted 3 minutes each and were separated by a figural fluency task (to minimize recency effects, to discourage verbal rehearsal, and to avoid interference of any verbal information with the learned verbal material) of 1 minute duration followed by a rest phase of 3 minutes duration. Blocks were separated by a rest phase (with eyes open) of 5 minutes duration, and a baseline recording (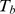) of 5 minutes was performed with eyes open preceding the experiment. In order to minimize possible order effects, we balanced the sequence of the blocks over subjects.

The number of correctly retrieved words (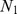 and 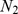; excluding perseverations) during the retrieval tasks served as measure of verbal memory (recall performance). Control subjects recalled 9.1±3.5 (range: 3–14) words (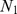) during 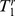, and 11.5±2.8 (range: 6–15) words (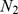) during 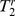. Epilepsy patients recalled 5.6±3.6 (range: 0–11) words (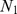) during 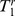, and 7.7±3.5 (range: 3–13) words (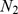) during 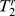. The correlation between 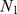 and 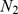 (Pearson's correlation coefficient; control subjects: 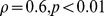; epilepsy patients: 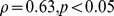) as well as the difference between the distributions of performance measures (t-tests; both groups: 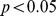) proved significant. Control subjects performed significantly better than epilepsy patients (t-tests; both tasks: 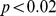), and both groups showed a significantly increased recall performance during 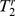 (t-tests; both groups: 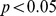).

During the whole examination procedure EEG data were acquired continuously at a sampling rate of 254.31 Hz (16 bit A/D conversion) within a bandwidth of 0–50 Hz from 29 electrodes, and right mastoid served as physical reference. Locations and nomenclature of these electrodes are standardized by the American Electroencephalographic Society [66]. In addition, we recorded right horizontal and vertical electrooculogram. In order to minimize the influence of technical (e.g., amplifier resets) and physiologic artifacts (e.g., eye movements/blinks or head movements) we applied a wavelet-based correction scheme as described in detail elsewhere [67].

Patients were investigated, on average, seven days prior to pre-surgical evaluation with blood levels of the anticonvulsant medication within the therapeutic range.

### Construction of functional networks

To construct functional networks from EEG data we associated network nodes with EEG electrodes (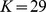) and inferred network links by estimating interdependencies between EEG time series from pairs of brain regions, regardless of their anatomical connectivity. For this purpose, and motivated by an increasing evidence for (phase) synchronization between distant brain areas to play an important role in learning- and memory-related processes [49], [68], [69], we employed the concept of time-variant phase coherence [70] which is an established method for studying time-variant changes in phase synchronization. The mean phase coherence [71]

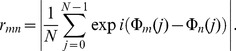
(1)is the temporal average of the differences of the instantaneous phases 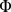 of time series from nodes 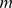 and 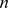, and 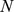 denotes the number of data points. By definition, 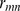 is confined to the interval [0,1] where 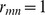 indicates fully phase-synchronized systems. We used the analytic signal approach [72], [73] with which instantaneous phases are obtained from the Hilbert transform of a time series. An important property of the analytic signal approach is that the the instantaneous frequency–in case of two or more superimposed oscillatory components–relates to the predominant frequency in the Fourier spectrum [74], [75] which may be subject to fluctuations in the time series. In such a case the instantaneous frequency varies rhythmically around the predominant frequency resulting in spurious estimates of the instantaneous phase. However, such effects can be reduced by taking the temporal average (cf. Eq. 1) or by applying adaptive approximation methods proposed recently [76]. From an electrophysiological point of view, we consider it more reasonable to look adaptively (via the Hilbert transform) at synchronization between predominant rhythms in the EEG than to look at synchronization in some a priori fixed frequency bands (e.g., via wavelet) for which there is no power in the time series (cf. Refs. [75], [77], [78]).

For further analyses and to allow an interval-based estimation of the mean phase coherence, we split the EEG time series into consecutive time intervals of 16.1 s duration each (corresponding to 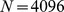 data points) and estimated, for each time interval, the elements 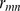 of the phase synchronization matrix **R**. From this matrix we constructed binary networks using a thresholding approach, and set the non-diagonal elements of the adjacency matrix **A** to 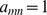 if the corresponding entry 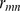 of **R** exceeded a threshold 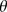, and to 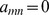 otherwise (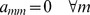). The choice of a threshold is not trivial, and there is currently no commonly accepted method to infer links from estimators of signal interdependence. However, since the number of nodes and the link density can affect network characteristics [79]–[85] precautions must be taken to minimize such influences when comparing between different networks. In order to determine 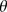 we requested that the networks are sparse (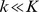) and that they do not disintegrate into unconnected network components as this leads to infinite values for the average shortest path length. Connectedness is guaranteed for mean degrees 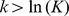, at least for random networks [86], and with the fixed number of nodes 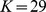 we obtained a mean degree 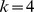, such that 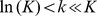 holds.

### Computation of statistical network characteristics

Here we considered the average shortest path length 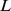 and the clustering coefficient 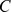 as global statistical characteristics of a functional brain network. We used an algorithm proposed in Ref. [87] to determine the shortest paths between all pairs of nodes from the adjacency matrix **A**. The length of the shortest path 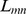 between two nodes 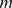 and 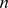 is the minimum number of links to traverse in order to get from 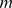 to 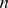 (or vice versa). With this definition we derived the average shortest path length 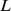 of the network by taking the average of all 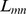 (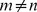) with 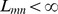. The clustering coefficient 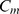 for node 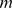 can be defined as (see, e.g., [1]): 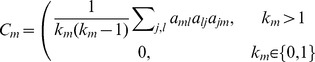
(2)where 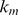 denotes the degree, i.e., the number of in- or out-going connections of node 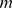. We averaged 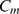 over all nodes to obtain the clustering coefficient 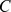 of a network.

In the following, we consider the baseline recording (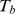) and the learning tasks (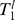 and 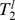) only. We omitted the retrieval tasks (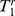 and 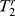) and the figural fluency task in order to avoid the influence of movement-related artifacts. With the interval-based estimation of interdependencies we obtained for each subject 11 intervals for 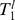 and for 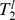 and 18 intervals for 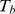. Averaging over intervals resulted in mean network characteristics which we denote as 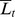 and 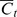 (the suffix 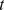 denotes task).

## Results

### Task-related modifications of functional brain networks

**Figure 2:**
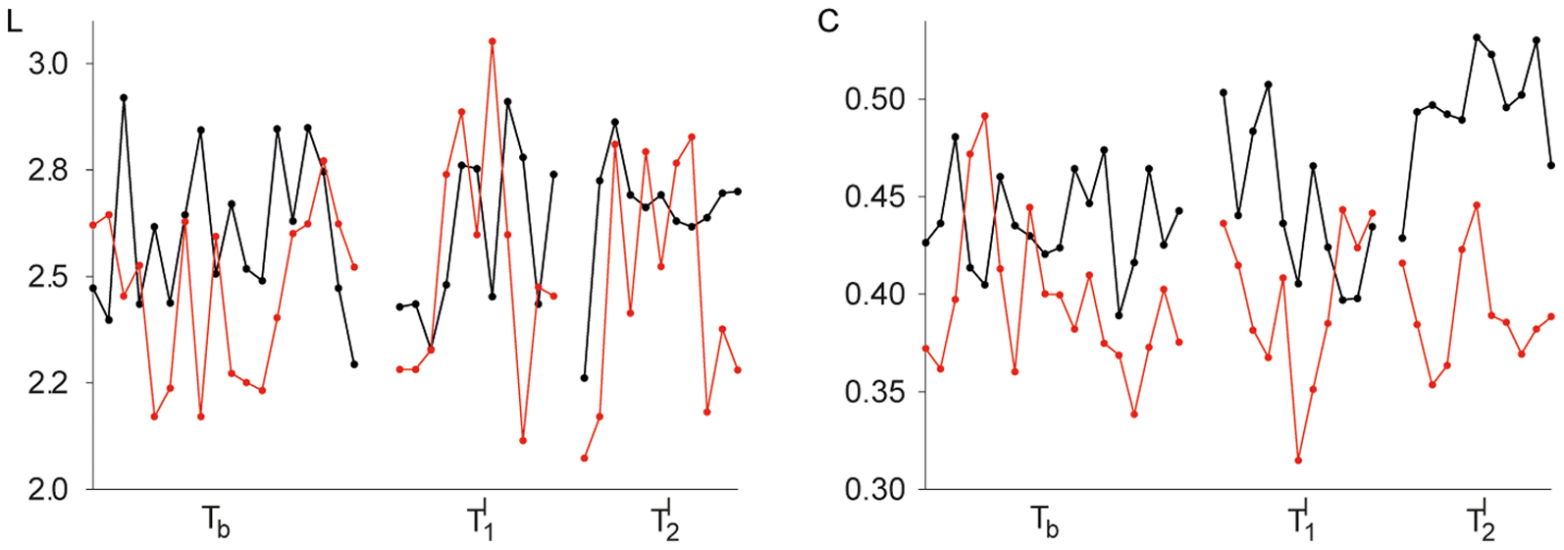
Exemplary temporal evolutions of global statistical network characteristics during different neuropsychological tasks. Time courses of average shortest path length 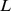 (left) and clustering coefficient 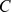 (right) from an epilepsy patient (red lines) and a control subject (black lines). Baseline recording (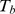); incidental learning task (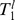); intentional learning task (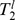). Lines are for eye-guidance only.

In Figure 2 we show, for an exemplary epilepsy patient and an exemplary control subject, temporal evolutions of the network characteristics during different neuropsychological tasks. Independent of tasks, both characteristics exhibited considerable fluctuations over time, and the patient's fluctuations were more pronounced. In both subjects, the learning tasks (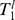 and 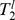) did not appear to modify the average shortest path length of the functional brain networks, as we could not observe clear-cut differences in 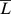 between any tasks. The same holds true for the clustering coefficient of the patient's functional brain network. For the control subject however, the intentional learning task (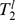) led to a significant increase of the clustering coefficient 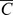 (when compared to both 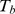 and 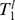; t-tests; 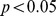). Both network characteristics attained slightly higher values in the control subject during all tasks. When comparing the characteristics' distributions from the patient group and the control group however, we could not observe any significant differences between the groups despite their significantly different recall performances. For the binary networks considered here, this observation is in line with our previous results [67]. In the following, we present our findings obtained from merged groups.

**Figure 3:**
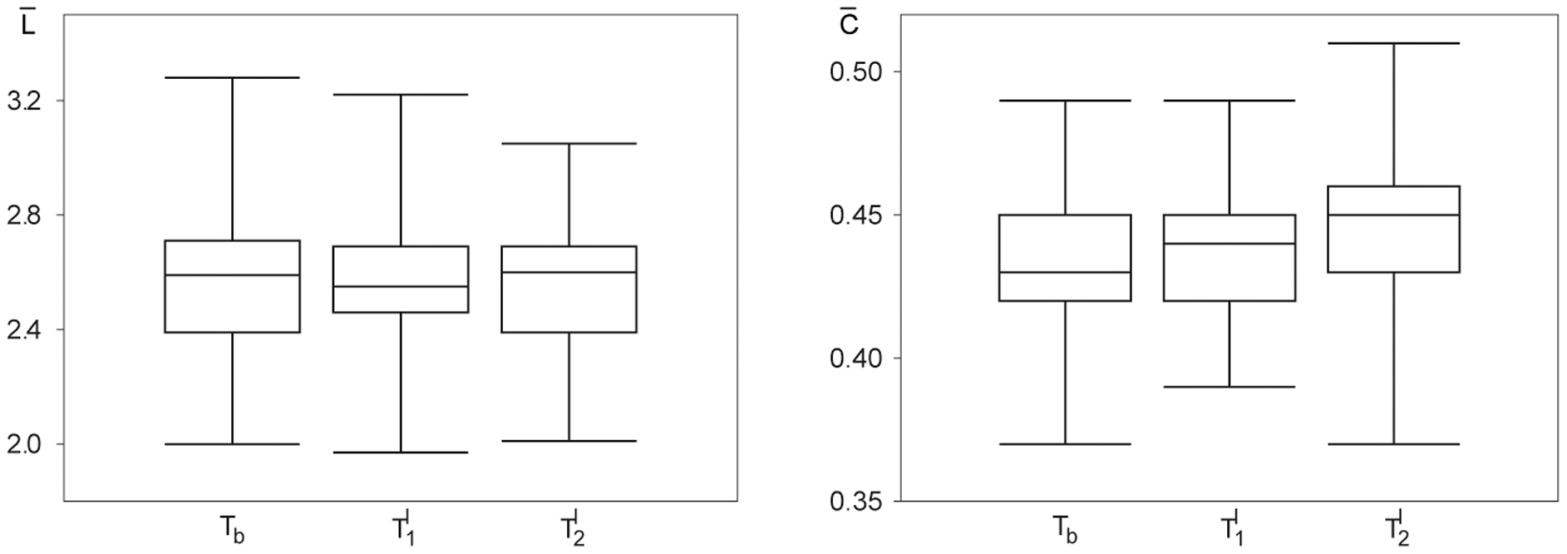
Comparison of the distributions of global statistical network characteristics from 33 subjects for different neuropsychological tasks. Box-plots of the average shortest path length 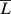 (left) and the clustering coefficient 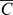 (right) for the baseline recording (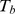) and for the incidental (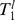) and the intentional learning task (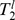). Bottom and top of a box are the first and third quartiles, and the band inside a box is the median. The ends of the whiskers represent the minimum and maximum of the data.

In order to investigate whether the aforementioned influences of neuropsychological tasks on network characteristics extend beyond exemplary data, we estimated–for all subjects and each task–statistical properties of the distributions of 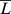 and 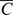 and evaluated possible differences between tasks (ANOVA with Greenhouse-Geisser and Huynh-Feldt corrections for departure from sphericity; 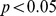). The distributions differed between tasks for the clustering coefficient but not for the average shortest path length. From the box-plots shown in Figure 3 it can be deduced that both learning tasks did not significantly modify the average shortest path length 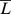 of the functional networks. The intentional learning task 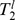, however, led to a small but significant increase of the networks' clustering coefficient (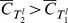 and 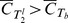; t-tests; 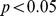).

### Relationship between network modifications and recall performance

We investigated whether network modifications induced during the learning tasks (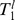 and 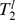) are related to the number of words (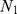 and 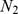) subjects recalled during the subsequent retrieval tasks (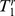 and 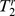). Given the high interindividual variability (cf. Figure 3) we focused on the relative deviation of the average shortest path length and of the clustering coefficient during the learning tasks from the respective values during the baseline recording 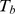, i.e. 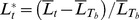 and 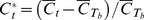, where 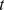 denotes 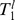 and 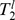, respectively.

**Figure 4:**
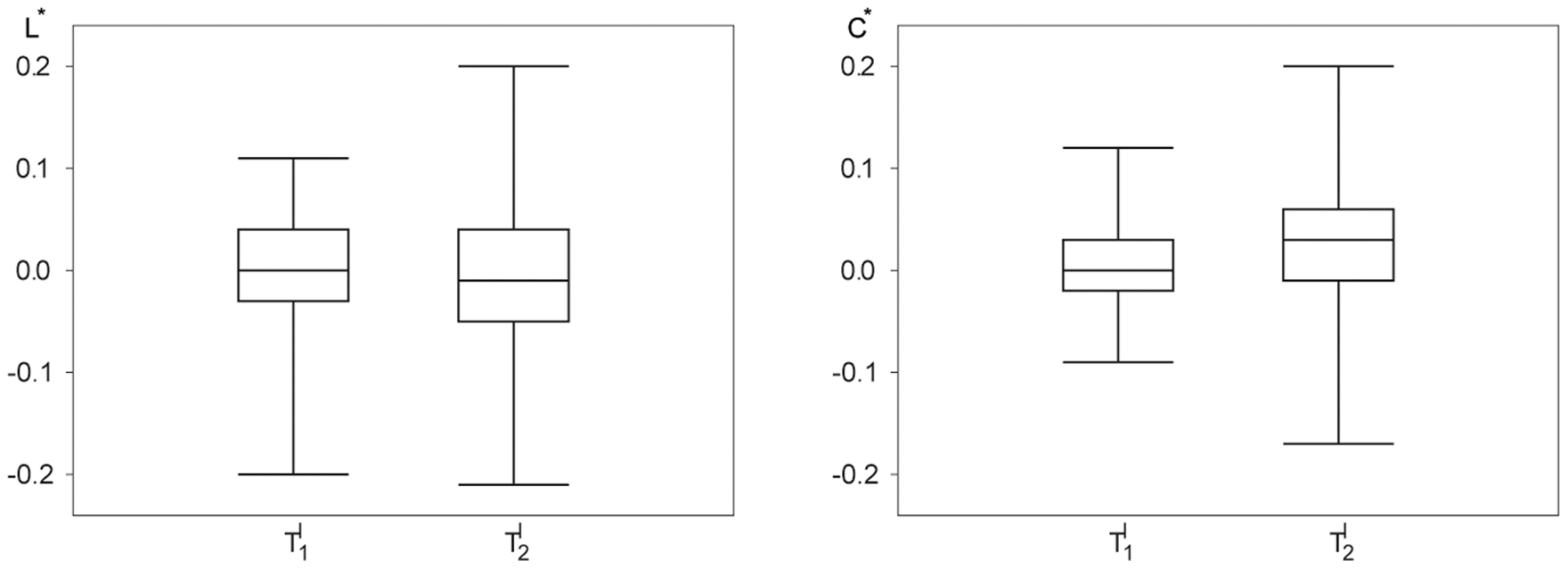
Comparison of the distributions of task-induced modifications of global statistical network characteristics. Box-plots (see Figure 3 for details) of the relative deviation of the average shortest path length 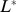 (left) and of the clustering coefficient 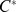 (right) during the learning tasks (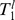 and 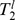) from the respective values during the baseline recording 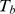.

**Figure 5:**
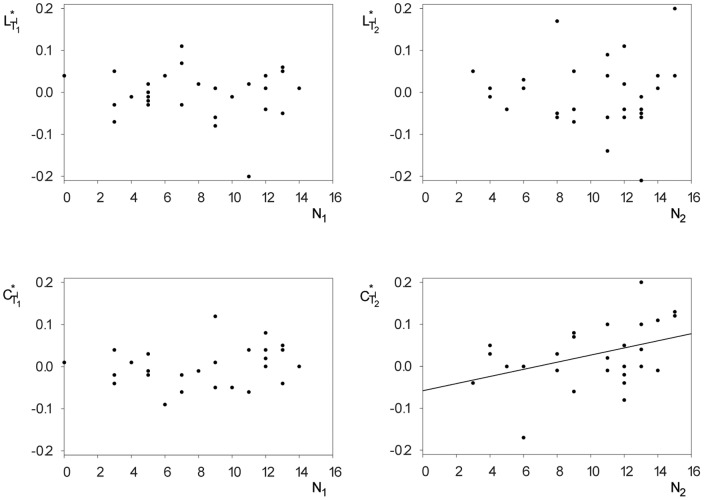
Relationships between task-induced modifications of global statistical network characteristics and recall performances. Scatterplots of relative deviations of the average shortest path length 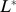 (top) and of the clustering coefficient 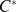 (bottom) during incidental 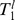 (left) and intentional learning 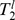 (right) from the respective values during the baseline recording and subsequent recall performances 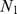 and 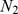. A significant correlation could only be observed between the relative clustering coefficient 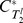 and the number 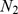 of recalled words during 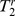 (linear regression is represented with a solid black line).

For the whole group of subjects, both characteristics deviated up to 20% from the baseline (cf. Figure 4). On average, however, deviations of the average shortest path length were close to zero (0.3% for 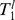; 0.2% for 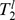; no significant difference), while those of the clustering coefficient amounted to 2.9% for 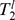, being significantly higher than the deviation for 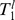 (0.2%; t-test; 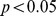). Interestingly, only for the intentional learning task–for which subjects were explicitly instructed to learn the presented material–could we observe a significant positive correlation (Pearson's correlation coefficient 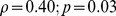; cf. Figure 5) between the relative clustering coefficient 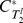 and the number 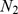 of subsequently retrieved words. Due to the aforementioned significant correlation between the recall performances, 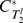 was also positively correlated to the number of words 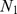 acquired during 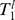 (Pearson's correlation coefficient 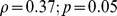). Of note, there were no significant correlations between the recall performances and the characteristics 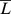 and 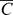 of the networks from the baseline recording 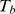.

## Conclusions

We investigated whether functional brain networks are differentially modified during incidental and intentional learning of verbal episodic material and whether modifications are related to subsequent recall performance. We assessed the two global statistical characteristics–clustering coefficient and average shortest path length–of functional brain networks that we derived from ongoing multichannel EEG data recorded from 33 subjects during a neuropsychological test schedule. Despite the fact that learning and memory-related processes involve structures of the lateral and mesial temporal lobes, whose dynamics may not be directly accessible with non-invasive EEG recordings, we observed differential learning-related modifications of the networks' global statistical properties. While there were no detectable modifications of the average shortest path length, networks attained a significantly higher clustering coefficient during intentional learning as compared to incidental learning and to the resting state. Interestingly, modifications during intentional learning even allowed us to predict the subsequent recall performance: the more pronounced the modifications of the clustering coefficient the higher the recall performance. An elevated clustering coefficient might be indicative of an increased occurrence of small but tightly connected groups of network nodes. Identification of these groups (or communities) using appropriate analysis techniques [88], [89] may aid in further exploration of the biological relevance of communities [29] and may provide novel insights into the relationship between the topology of functional brain networks and cognitive functions.

Our findings are in line with some previous reports on task-related modifications of global properties of functional brain networks and their relationship to task performance (e.g. [19], [24], [29], [35]) and support the view that higher cognitive functions–such as learning- and memory-related processes–result from dynamic interactions of distributed brain areas operating in large-scale networks. The differences seen for intentional and for incidental learning, however, indicate that task-induced topological changes of functional brain networks may not only reflect the cognitive process per se but also attentional effects [90]–[92]. Exploring the interactions between attention-related networks and those important for learning and memory remains a challenging task.
